# Data on shale-water based drilling fluid interaction for drilling operation

**DOI:** 10.1016/j.dib.2018.06.014

**Published:** 2018-06-21

**Authors:** Emeka Emmanuel Okoro, Kevin C. Igwilo, Angela Onose Mamudu, Evelyn Bose Ekeinde, Adewale Dosunmu

**Affiliations:** a Petroleum Engineering Department, Covenant University Ota, Nigeria; b Petroleum Engineering Department, Federal University of Technology, Owerri, Nigeria; c Chemical Engineering Department, Covenant University Ota, Nigeria; d Petroleum Engineering Department, University of Port Harcourt, Nigeria

## Abstract

The shale dispersion test (rolling test) is a common procedure that is used to measure the interactions between drilling fluids and shales. The shale rolling test depends on the moisture content of the shale, the shale composition, the viscosity of the test fluid, the rotation speed of the rollers, and the test temperature. The rheological behavior of the test fluid has the strongest influence on test results. The data was generated experimentally, shale samples from Agbada formation Niger-Delta was used. These shale samples were cored at a depth of 2000 ft and 3400 ft. Water based mud that will minimize shale dispersion and swelling of shale was formulated. The dispersion test was conducted, and it involves exposing a weighted quantity of sized shale to the formulated mud in roller-oven. This test is used to design fluids and screen the effectiveness of inhibitor additives to maintain the integrity of the cuttings and minimize the interaction of fluids with the shale sections during the drilling and completion operations. The swelling test was conducted and the linear expansion adopted because it is the most representative of the increase seen by the wellbore but was measured in the direction perpendicular to the bedding plane as this is the direction of swelling into the wellbore.

**Specifications Table**

**Table:** 

Subject area	Petroleum Engineering
More specific subject area	Drilling Engineering
Type of data	Table, figure
How data was acquired	Experimental
Data format	Raw, Analyzed
Experimental factors	The shale rolling test depends on the moisture content of the shale, the shale composition, the viscosity of the test fluid, the rotation speed of the rollers, and the test temperature.
Experimental features	The rolling test is a useful technique to measure the interactions of electrolytes with shale granules in partially formulated drilling fluids
Data source location	Rivers State, Nigeria.
Data accessibility	Data are available within this article.
Related research article	None.

**Value of the data**

• These data describe the rheological behavior of the test fluid as the strongest influence on the test results.
• The data can be used to design fluids and screen the effectiveness of inhibitor additives to maintain the integrity of the cuttings.
• These data can be applied when analyzing the interaction of fluids with the shale sections during drilling operations.
• The data analyzed the behavior of drilling cuttings in drilling fluids as they travel up the annulus.

## 1 Data

Dispersion tests were carried out using various drilling fluids for Shale samples in Well A and B at 120 °F. Percentage recovery, a measure of shale recovered after dispersion tests were calculated. Table 1 shows the percent recoveries of Shale in Well A for the bentonitic fluids. Maximum recovery was obtained at 120 °F using a fluid composition of 3% Bentonite+1% KCl followed by 3% Bentonite+1% CaCl_2_.

**Table 1:** Well A percentage recoveries for bentonitic fluids.

**Fluid composition**	**% Recovery**
Base Fluid	47.40
3% Bentonite+0.3% KCl	69.89
3% Bentonite+1% KCl	79.67
3% Bentonite+0.3% CaCl_2_	70.56
3% Bentonite+1% CaCl_2_	77.15
3% Bentonite+0.3% NaCl	66.34
3% Bentonite+1% NaCl	76.83

Table 2 shows the percent recoveries of Shale sample in Well B for the bentonitic fluids. Maximum recovery was obtained at 120°F using a fluid composition of 3% Bentonite+1% KCl followed by 3% Bentonite+1% CaCl_2_. The results are in agreement with the literature, that K^+^ and Ca^2+^ ions are added to the water-based muds to inhibit the clay from dispersing, to stop it from breaking up when attacked by an aqueous solution [1], [2]. These ions commonly replace the sodium ion (Na^+^) associated with the clay in the shale, creating a more stable rock that is able to resist hydration. The results show that the dispersed solids in the fluids were large. The dispersion is a function of changes in the structure of the shale matrix and in the bound and crystalline water caused by the hydration of the cores [3], [4].

**Table 2:** Well B percentage recoveries for bentonitic fluids.

**Fluid composition**	**% Recovery**
Base Fluid	50.40
3% Bentonite+0.3% KCl	70.40
3% Bentonite+1% KCl	77.20
3% Bentonite+0.3% CaCl_2_	66.80
3% Bentonite+1% CaCl_2_	71.80
3% Bentonite+0.3% NaCl	65.80
3% Bentonite+1% NaCl	70.80

The Well A cored at a depth of 2000 ft, is made up of 20% Quartz and 52% Clay. It contains other non-clay minerals such as feldspar and carbonates in minimal amounts. The bulk of the clay content consists of illite and mixed clays with a small amount of smectite. The existence of smectite indicates the probability of some swelling and dispersion in aqueous solution. Well B cored at a depth of 3400 ft, is composed of 22% Quartz and 51% Clay. It also contains negligible amount of non-clay minerals such as feldspar and carbonates. Zero smectite levels indicate low swelling tendencies.

The results are in agreement with the literature, that K^+^ and Ca^2+^ ions are added to the water-base muds to inhibit the clay from dispersing, to stop it from breaking up when attacked by an aqueous solution. These ions commonly replace the sodium ion (Na^+^) associated with the clay in the shale, creating a more stable rock that is able to resist hydration. The results show that the dispersed solids in the fluids were large. The dispersion is a function of changes in the structure of the shale matrix and in the bound and crystalline water caused by the hydration of the cores.

The plastic viscosity (PV) and yield point (YP) were calculated before and after dispersion. Table 3, Table 4, Table 5, Table 6 show the PV and YP values obtained for the bentonitic fluids.

**Table 3:** Plastic viscosity values for the bentonitic fluids before and after hot rolling with shale in Well A.

**Fluid composition**	**Plastic viscosity (cp)**	
	**At 120 °F**	
	**Before**	**After**
3% Bentonite+1% KCl	5	7
3% Bentonite+0.3% KCl	7	8
3% Bentonite+1% CaCl_2_	4	10
3% Bentonite+0.3% CaCl_2_	5	8
3% Bentonite+1% NaCl	5	6
3% Bentonite+0.3% NaCl	5	6

**Table 4:** Yield point values for bentonitic fluids before and after hot rolling with shale in Well A.

**Fluid composition**	**Yield point (lb/100 ft**^**2**^**)**	
	**At 120 °F**	
	**Before**	**After**
3% Bentonite+1% KCl	5	8
3% Bentonite+0.3% KCl	5	9
3% Bentonite+1% CaCl_2_	24	19
3% Bentonite+0.3% CaCl_2_	4	8
3% Bentonite+1% NaCl	4	7
3% Bentonite+0.3% NaCl	6	10

**Table 5:** Plastic viscosity values for bentonitic fluids before and after hot rolling with shale in Well B.

**Fluid composition**	**Plastic viscosity (cp)**	
	**At 120 °F**	
	**Before**	**After**
3% Bentonite+1% KCl	5	8
3% Bentonite+0.3% KCl	7	9
3% Bentonite+1% CaCl_2_	4	9
3% Bentonite+0.3% CaCl_2_	5	8
3% Bentonite+1% NaCl	5	6
3% Bentonite+0.3% NaCl	5	8

**Table 6:** Yield point values for bentonitic fluids before and after hot rolling with shale in Well B.

**Fluid composition**	**Yield point (lb/100 ft**^**2**^**)**	
	**At 120 °F**	
	**Before**	**After**
3% Bentonite+1% KCl	5	4
3% Bentonite+0.3% KCl	5	6
3% Bentonite+1% CaCl_2_	24	19
3% Bentonite+0.3% CaCl_2_	4	5
3% Bentonite+1% NaCl	4	5
3% Bentonite+0.3% NaCl	6	5

A comparison of PV values before and after hot-rolling the shale samples showed distinct outcome with the salt/bentonite fluids. The salt/bentonite fluids showed an increase in PV when hot-rolled at 120 °F; which indicates shale dispersion. Yield point for the salt/bentonite fluids showed a decrease in YP after hot-rolling at 120 °F. YP is a function of electrostatic forces between fluid particles in motion [5]. For Bingham fluids, it is also the shear stress required to initiate flow in fluids. From YP results, the dispersion tests reduced the attractive force between the solid particles significantly.

Further analysis of the dispersion tests involved the measurement of the fluid rheology before and after the tests. Values of shear stress (τ) and shear rate (γ) were calculated and presented in Table 7, Table 8, Table 9.

**Table 7:** Shear stress and shear rate of bentonitic fluids before dispersion tests at 120 °F.

Shear rate(1/s)	Shear stress of 3% Bentonite+1% KCl	Shear stress of 3% Bentonite+0.3% KCl	Shear stress of 3% Bentonite+1% CaCl_2_	Shear stress of 3% Bentonite+0.3% CaCl_2_	Shear stress of 3% Bentonite+1% NaCl	Shear stress of 3% Bentonite+0.3% NaCl
1021.8	0.1599	0.2025	0.3411	0.1492	0.1492	0.1706
510.9	0.1066	0.1279	0.2985	0.0959	0.0959	0.1173
340.6	0.0853	0.1066	0.2878	0.0746	0.0746	0.0959
170.3	0.0533	0.0746	0.2665	0.0533	0.0533	0.0640
102.2	0.0426	0.0533	0.2558	0.0320	0.0426	0.0533
51.09	0.0320	0.0426	0.02345	0.0267	0.0320	0.0426

**Table 8:** Shear stress and shear rate of bentonitic fluids after dispersion tests with shale from Well A at 120 °F.

Shear rate(1/s)	Shear stress of 3% Bentonite+1% KCl	Shear stress of 3% Bentonite+0.3% KCl	Shear stress of 3% Bentonite+1% CaCl_2_	Shear stress of 3% Bentonite+0.3% CaCl_2_	Shear stress of 3% Bentonite+1% NaCl	Shear stress of 3% Bentonite+0.3% NaCl
1021.8	0.2345	0.2665	0.4157	0.2558	0.2025	0.2345
510.9	0.1599	0.1812	0.3091	0.1706	0.1386	0.1706
340.6	0.1279	0.1492	0.2878	0.1279	0.1066	0.1173
170.3	0.0959	0.1066	0.2239	0.1066	0.0746	0.0746
102.2	0.0640	0.0959	0.2025	0.0746	0.0533	0.0640
51.1	0.0426	0.0640	0.1812	0.0533	0.0426	0.0426

**Table 9:** Shear stress and shear rate of bentonitic fluids after dispersion tests with shale from Well B at 120 °F.

Shear rate(1/s)	Shear stress of 3% Bentonite+1% KCl	Shear stress of 3% Bentonite+0.3% KCl	Shear stress of 3% Bentonite+1% CaCl_2_	Shear stress of 3% Bentonite+0.3% CaCl_2_	Shear stress of 3% Bentonite+1% NaCl	Shear stress of 3% Bentonite+0.3% NaCl
1021.8	0.2132	0.2558	0.3944	0.2239	0.1812	0.2239
510.9	0.1279	0.1599	0.2985	0.1386	0.1173	0.1386
340.6	0.1066	0.1279	0.2558	0.1066	0.0853	0.1066
170.3	0.0640	0.0853	0.2132	0.0746	0.0533	0.0640
102.2	0.0426	0.0640	0.1919	0.0533	0.0426	0.0426
51.1	0.0320	0.0426	0.1706	0.0320	0.0320	0.0320

## 2 Experimental design, materials, and methods

The dispersion test involves exposing a weighed quantity of sized shale pieces (2–4 Sieve Opening Millimeters) to a formulated fluid in a conventional roller-oven cell. The test provides long-term exposure of the shale to the fluid under mild agitation. Under such conditions, dispersion of the shale into the fluid will occur depending on the tendency of the shale to disperse and the inhibitive properties of the fluid. Shale dispersion is a process by which shale cuttings disintegrate into smaller sizes. It is a function of mechanical factors such as shear and chemical factors such as hydration (Guizhong et al. [6]). The rheological characteristics of the fluid also can influence the test results by altering the amount of agitation in the rolling phase. The fluid and shale are rolled together in a roller oven for 16 hours at 120 °F. After cooling to room temperature, the fluid is poured over a 0.023 mesh size and the retained shale pieces are recovered, washed, weighed, and dried overnight at 200 °F. Afterward, the sample is re-weighed to determine the percent recovery [7].

Fluid rheological properties were also measured using a six-speed Fann 35 viscometer before and after dispersion tests. This was done in order to monitor rheological alterations due to shale disintegration and temperature changes after dispersion test was carried out. Values of shear stress (τ) and shear rate (γ) were calculated using the following equations:

For shear stress,

(1) $$\tau =0.01066\times \theta_{i}\times \;\;N$$

where

*τ*=shear stress (lb_f_/ft^2^)
*θ_i_*=dial reading at ith rpm
*N*=spring factor=1
For shear rate,

(2) γ=1.703×RPM

where

*γ*=shear rate (1/s)
RPM=viscometer rotational speed

Salt additives {Potassium Chloride (KCl), Sodium Chloride (NaCl), Calcium Chloride (CaCl_2_)}, when used in the drilling fluid have proved to be beneficial in stabilizing shale formations. These additives lower the water activity of the drilling fluid, which leads to higher osmotic pressure gradients. Also, solute mobility is low in shale, which increases the membrane efficiency of the shale/drilling fluid system. As a result, the effective osmotic pressures generated could be strong enough to offset the hydraulic mud over-balance, which indeed could lead to shale stabilization through dehydration. Also, the main function of viscosifier is to increase the viscosity of the mud filtrate, and thus, reduce the hydraulic flow and pressure penetration into shale formations. They also lower the water activity of the drilling fluid and thus generate osmotic potentials [8]. These two factors may lead to shale stabilization.

Linear Swelling tests were carried out on Shale samples from Well A and B using Water Base-mud and deionized water. Both shale samples swell in deionized water after immersion for 24 h. They also showed reactivity when placed in all the test fluids. Maximum linear swell for this shale was obtained in the deionized water.
