# Supplementary material for: Data on shale-water based drilling fluid interaction for drilling operation
Source: Data Brief. 2018 Jun 21;19:1620–6. doi: 10.1016/j.dib.2018.06.014 (PMC6141129; doi:10.1016/j.dib.2018.06.014)
Supplement: Supplementary file 1 — Supplementary material [file mmc1.doc]

May 29, 2018

Manuscript No. DIB-D-18-01097

Dear Managing Editor,

There is no concern raised about the reviewer’s comment. All issues requested and identified have been effected and highlighted in the reversed manuscript.

The authors appreciate the contributions and state that there is no conflict of interest.

I look forward to hearing from you soon.

Yours sincerely,

Dr. Okoro Emeka Emmanuel

FOR: The Authors
